# Morel Production Related to Soil Microbial Diversity and Evenness

**DOI:** 10.1128/Spectrum.00229-21

**Published:** 2021-10-13

**Authors:** Hao Tan, Tianhai Liu, Yang Yu, Jie Tang, Lin Jiang, Francis M. Martin, Weihong Peng

**Affiliations:** a Institute of Agricultural Resources and Environments, Sichuan Academy of Agricultural Sciences, Chengdu, China; b School of Bioengineering, Jiangnan University, Wuxi, China; c Scientific Observation and Experimental Station of Agro-microbial Resource and Utilization in Southwest China, Ministry of Agriculture and Rural Affairs, Chengdu, China; d Université de Lorraine, Institut National de la Recherche Agronomique, UMR Interactions Arbres/Microorganismes, Centre INRA-GrandEst-Lorraine, Champenoux, France; e Beijing Advanced Innovation Center for Tree Breeding by Molecular Design, Beijing Forestry University, Beijing, China; University of Melbourne

**Keywords:** morel, large-scale farming, fructification, soil microbial diversity, community evenness, predominant fungi

## Abstract

Black morel is a widely prized ascomycetous mushroom with culinary value. It was once uncultivable but can now be cultivated routinely in ordinary farmland soils. Large-scale morel farming sometimes encounters nonfructification for unknown reasons. In spring 2020, many morel farms in the area of Chengdu-Plain, China, exhibited no fructification at all, causing disastrous economic loss to the farmers. To determine potential ecological factors associated with the different performance of morel production in these farms, 21 affected sites versus 11 sites with normal fructification performance were analyzed to compare soil microbiota and physiochemical characteristics during fructification. The results indicated that soil physiochemical characteristics were unlikely to be a major reason for the difference between successful fructification and nonfructification. The soils with successful fructification had significantly higher diversity in both the fungal and bacterial communities than those with nonfructification. Morel yield was positively correlated with the α-diversity of fungal communities. The higher diversity of the successfully fructified soils was contributed by community evenness rather than taxonomic richness. In contrast, most nonfructification soils were dominated by a high proportion of a certain fungal genus, typically *Acremonium* or *Mortierella*, in the fungal communities. Our findings demonstrate the importance of microbial ecology to the large-scale agroindustry of soil-cultivated mushrooms.

**IMPORTANCE** Saprotrophic mushrooms cultivated in soils are subject to complex influences from soil microbial communities. Research on growing edible mushrooms has revealed connections between fungi and a few species of growth-promoting bacteria colonizing the mycosphere. The composition and diversity of the whole microbial community may also have an influence on the growth and production of soil-saprotrophic mushrooms. Morel mushrooms (*Morchella* spp.) are economically and culturally important and are widely prized throughout the world. This study used the large-scale farming of morels as an example of an agroecosystem for soil-saprotrophic mushroom cultivation. It demonstrated a typical pattern of how the microbial ecology in soil agroecosystems, especially the α-diversity level and community evenness among soil fungal taxa, could affect the production of high-value cash crops and the income of farmers.

## INTRODUCTION

**FIG 1:**
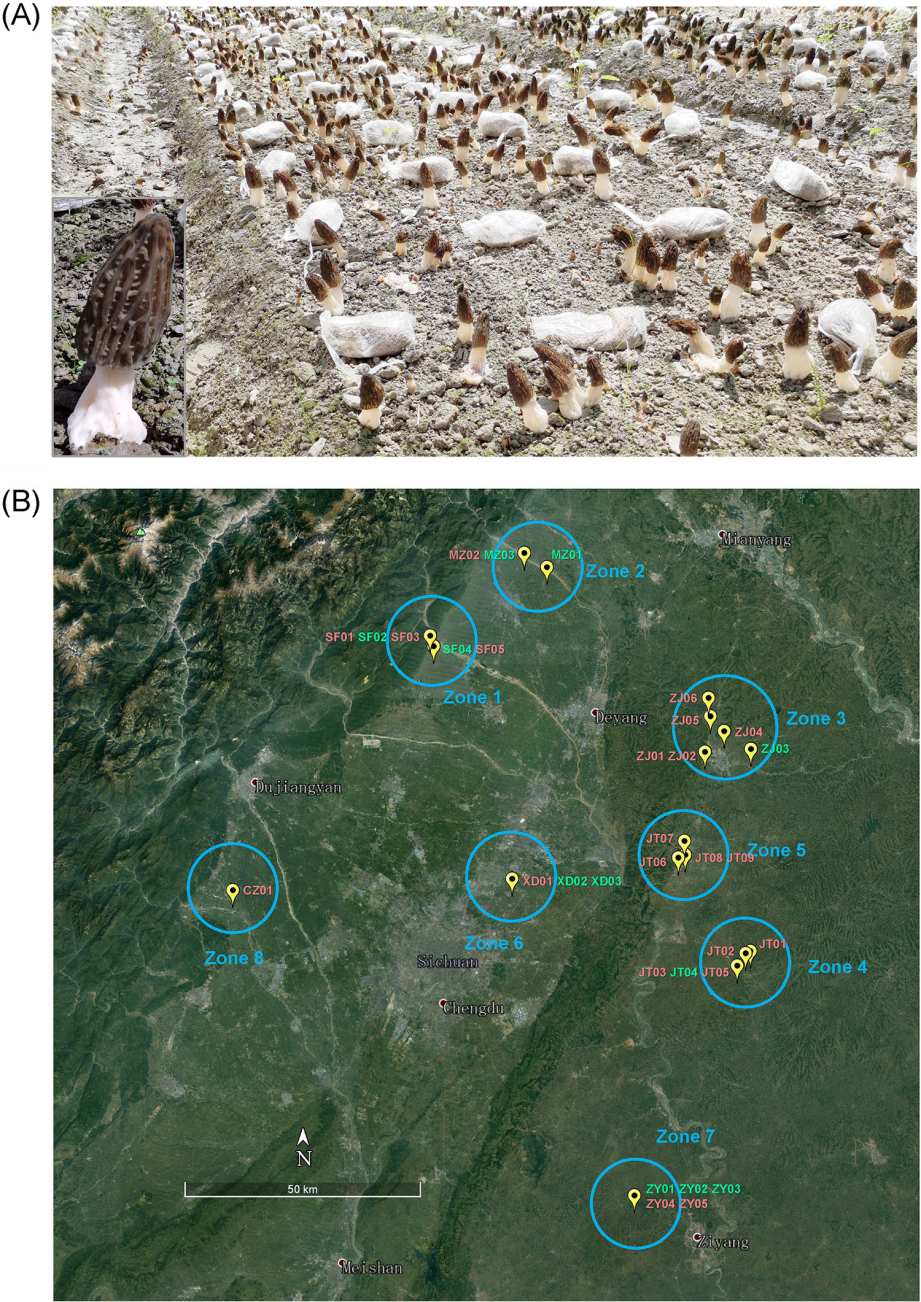
(A) Large-scale morel cultivation, with a magnified portrait of a morel ascocarp. (B) Geographic distribution of the 21 morel farms that exhibited nonfructification (red) and 11 with normal fructification performance (green).

Species in the ascomycetous genus *Morchella*, commonly known as morels, are highly prized gourmet mushrooms and culinary delicacies. Black morels are soil-saprotrophic members in the Elata clade of the *Morchella* genus (1), including Morchella importuna M. Kuo, O’Donnell & T.J. Volk, M. sextelata M. Kuo, and M. eximia Boud (2). These mushrooms were uncultivable wild mushrooms for most of their history (3, 4). In recent years, black morels have been domesticated as mushroom crops for large-scale production that can be cultivated routinely with a high yield in ordinary agricultural soils (5) (Fig. 1A). Although the area of black morel agroindustry is expanding rapidly both in China and around the world (2, 6, 7), morel farms sometimes encounter severe loss in the fruiting-body yield for unknown reasons. In the worst cases, farms that are trying to cultivate morels may not obtain any morel production over large areas. The phenomenon is suspected by morel growers to be an intrinsic instability of the present techniques for morel cultivation, which is severely hampering the development of the morel agroindustry.

Possible reasons leading to failure of morel fructification include low vitality of the cultivated strain (the so-called mushroom spawn) used, improper field management practices that provide unfavorable environmental conditions, and soilborne antagonists against the morel. Previous studies reported that the morel was vulnerable to a series of soilborne pathogens (8–11). The fructification yield of black morel was found to have an association with the community profiles of soil bacteria as well as the physiochemical characteristics of metal elements (12). A similar phenomenon was observed in the indoor production of Morchella rufobrunnea, which belongs to another clade of *Morchella* and is called the red-brown blushing morel (13). Fructifying beds with failed fructification were colonized by a high proportion of *Cephalotrichum* fungi (14), commonly known as nonpathogenic. Indeed, growing edible fungi requires an understanding of environmental microorganisms (15). It is hypothesized that the diversity of the soil microbial community might play an important role that influences the fructification of morels.

In the early spring of 2020, after a warm winter in the Chengdu-Plain area of Sichuan Province, China, many morel farms encountered severe or even complete failure of fructification, leading to disastrous economic loss to the farmers. The aim of this study was to determine the potential relationship between the soil microbial community and the performance of morel production on these farms. Soil physiochemical characteristics and soil microbial communities were analyzed during the fructification season to compare soils with successful fructification and those with no fructification.

## RESULTS

### Soil physiochemical characteristics.

In this study, a total of 128 soil samples from 32 sites were analyzed. Each site contained four plots, and each plot sample was composed of a mixed composite of 36 soil cores (see “Soil sampling” in Materials and Methods). Among the 32 morel farms investigated, 5 were located in Shifang (SF), 3 in Mianzhu (MZ), 5 in Zhongjiang (ZJ), 9 in Jintang (JT), 3 in Xindu (XD), 5 in Ziyang (ZY), and 1 in Chongzhou (CZ) (Fig. 1B; see also Table S1 in the supplemental material). Twenty-one of the 32 farms (SF01, SF03, SF05, MZ02, ZJ01, ZJ02, ZJ04, ZJ05, ZJ06, JT01, JT02, JT03, JT05, JT06, JT07, JT08, JT09, XD01, ZY04, ZY05, and CZ01) had no fructification (NF). In total, 150.42 ha harvested no morel at all. Eleven farms (SF02, SF04, MZ01, MZ03, ZJ03, JT04, XD02, XD03, ZY01, ZY02, and ZY03) fructified successfully (F), with a total of 32.18 ha. The estimated yield in the fructified farms varied between 0.15 and 0.6 kg m^−2^, with a weighted average of 0.42 kg m^−2^.

**FIG 2:**
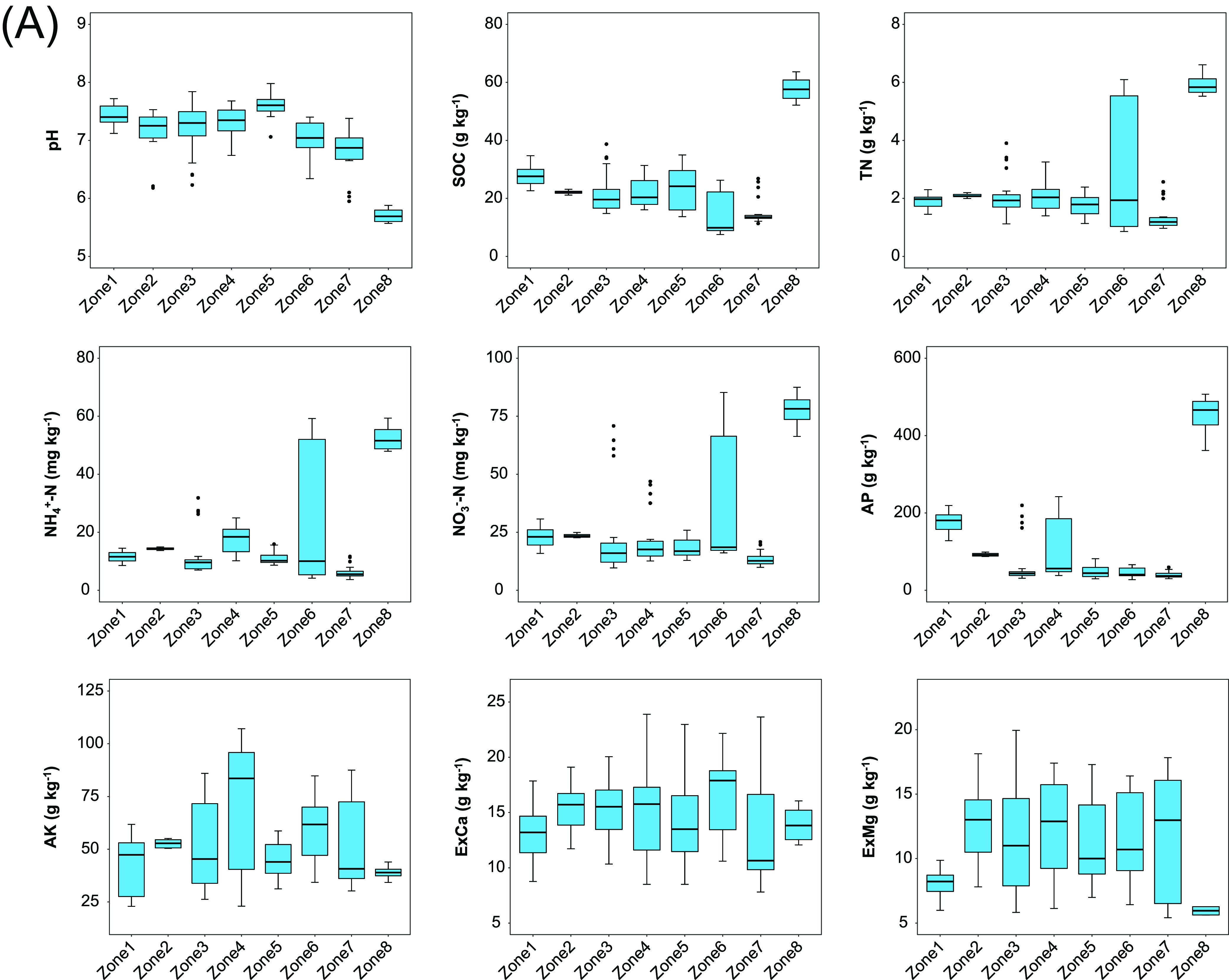

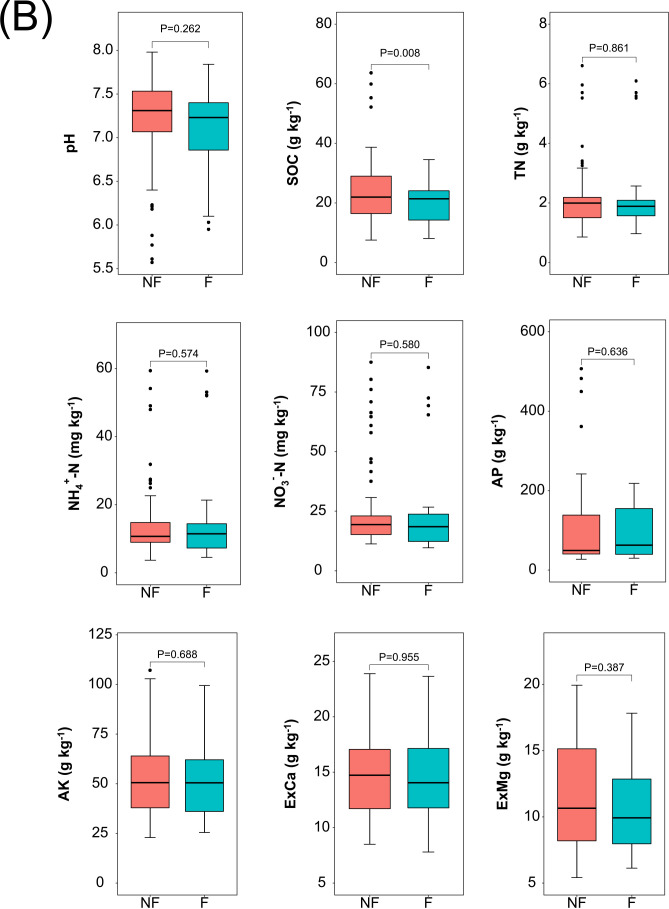

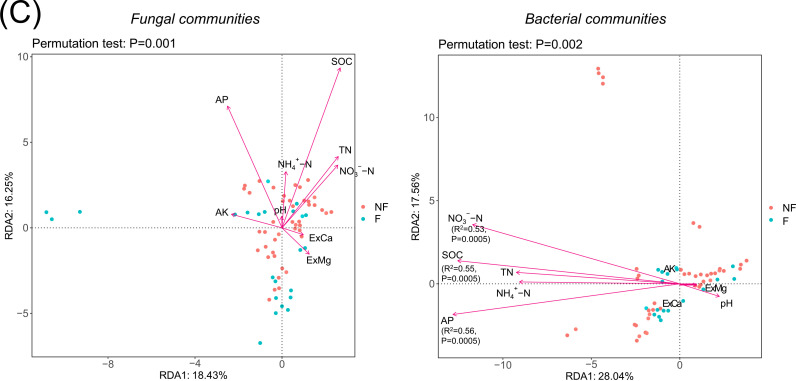
(A) Boxplots of the soil physiochemical characteristics of the eight zones. All the *P* values between different zones are provided in the supplemental material (Table S2). (B) Boxplots of the soil physiochemical characteristics, compared between the sites with successful fructification (F) and those with no fructification at all (NF). (C) Correlation between the microbial community profiles and the soil physiochemical characteristics, shown by redundancy analysis (RDA) plots. *R*^2^ and *P* values are labeled only when *R*^2^ is >0.5 and *P* is <0.001.

The 32 farms were divided into eight zones according to their clustering on the map (Fig. 1B). The distribution of the nonfructification sites was not geographically regular. The eight zones showed some differences in the soil physiochemical characteristics of pH, soil organic carbon (SOC), total nitrogen (TN), ammonium N (NH_4_^+^-N), nitrate N (NO_3_^−^-N), available P (AP), available potassium (AK), exchangeable Ca (ExCa), and exchangeable Mg (ExMg) (Fig. 2A). The ExCa, ExMg, and AK were mostly constant among the eight zones, while the SOC and AP were more variable. In particular, the soils of CZ01 with relatively lower pH and richer nutrients of C, N, and P were unique compared with the other samples. This reflected the variation of soil physiochemical characteristics between different locations. A comparison between the sites with successful fructification of morels versus those with no fructification at all revealed no significant difference in most of the examined soil physiochemical characteristics (Fig. 2B), except for the SOC, which was at a significantly higher level in the nonfructification sites than the fructified sites (*P* = 0.008). This may have been because the SOC in the fructification soils had been consumed to compose the biomass of morel ascocarps, which was removed by the harvest (5), whereas the SOC in the nonfructification soils remained. The results indicated that the soil physiochemical characteristics were unlikely to be a decisive factor to determine the fate of morel fructification. Furthermore, the redundancy analysis (RDA) plots showed that the fungal communities had weak correlations with the soil physiochemical characteristics (all *R*^2^ < 0.5) (Fig. 2C). This suggested that the soil fungal communities of different sites were unlikely to be shaped by the soil physiochemical characteristics. In comparison, the bacterial communities were mildly correlated with the AP, SOC, and NO_3_-N (0.5 < *R*^2^ < 0.6; *P* < 0.001).

### Microbial community diversity and evenness.

**FIG 3:**
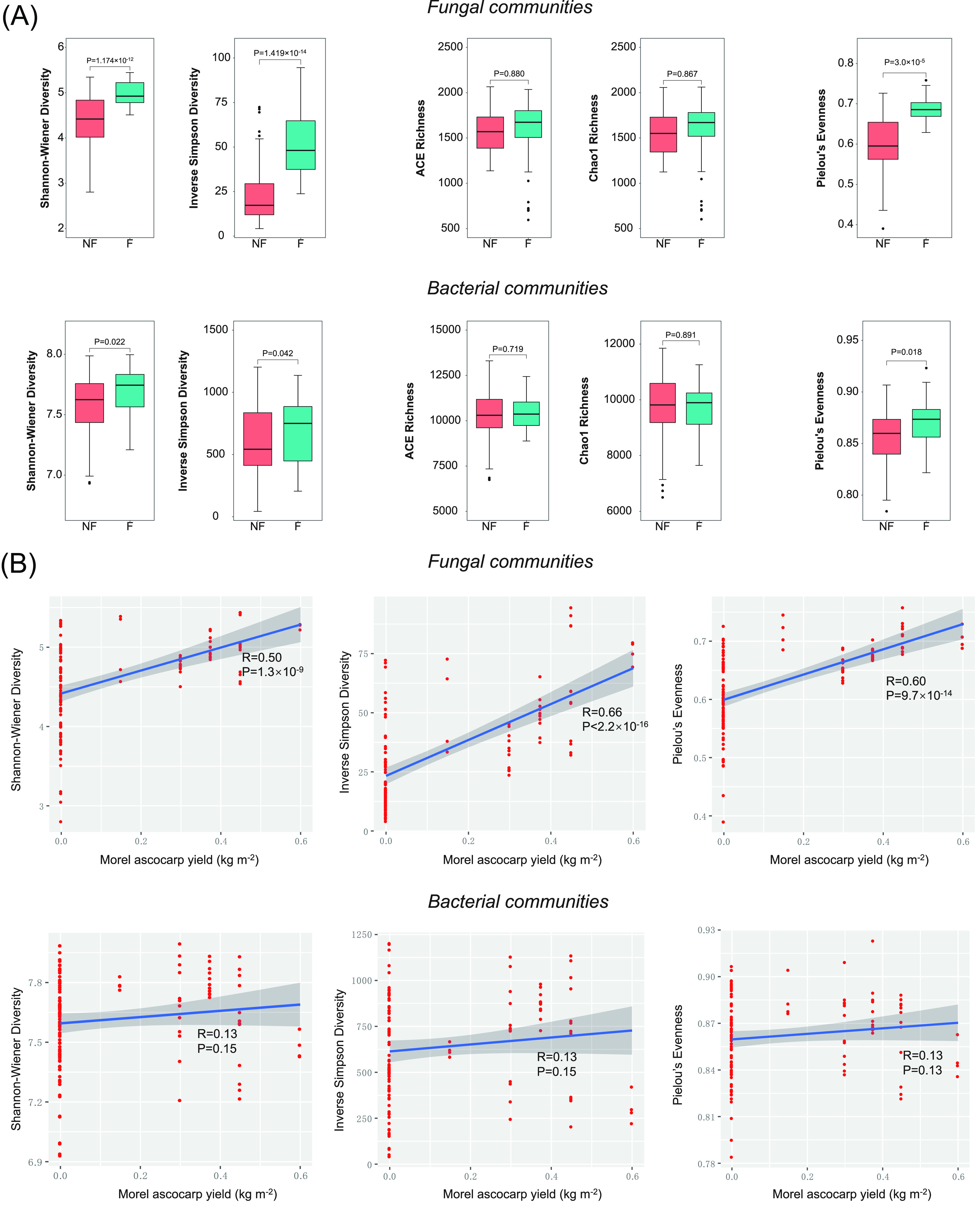

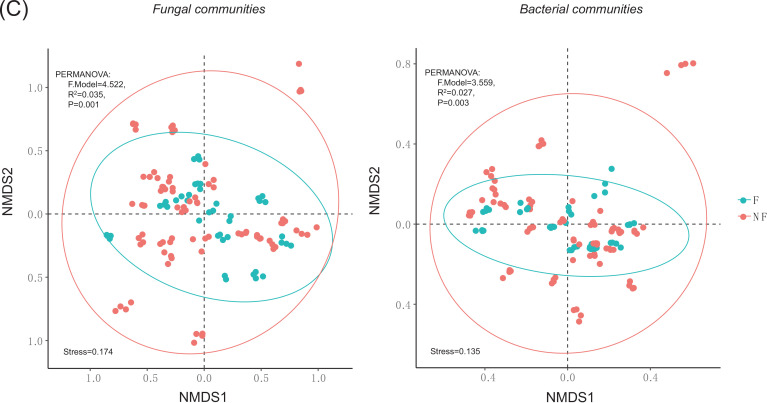
(A) α-Diversity estimators of the soil fungal and bacterial communities of the investigated morel farms. (B) Correlation between the yield level of morel ascocarps and the α-diversity estimators of the Shannon-Wiener index, the inverse Simpson’s index, and Pielou’s evenness index. (C) β-Diversity of the fungal and bacterial community profiles of the investigated morel farms, shown by nonmetric multidimensional scaling (NMDS) scattering plots.

The α-diversity of fungal and bacterial communities in the soils of the investigated morel farms was estimated by the following indices: the Shannon-Wiener and inverse Simpson’s diversity indices, measuring both community evenness and taxonomic richness; the ACE and Chao1 richness indices, measuring only taxonomic richness; and Pielou’s evenness index, measuring only community evenness. By comparison, the Shannon-Wiener and inverse Simpson’s diversity indices as well as Pielou’s evenness index were significantly higher in the fructified sites than the nonfructification ones for both the fungal and the bacterial communities (Fig. 3A). This trend was much more prominent in the fungal communities (*P* < 3.0 × 10^−5^) than in the bacterial communities (0.01 < *P* < 0.05). However, the taxonomic richness had no significant difference between nonfructification and fructification sites (*P* > 0.05) (Fig. 3A).

Because the α-diversity estimators of the Shannon-Wiener and the inverse Simpson’s diversity indices as well as the Pielou’s evenness index showed significant differences between the nonfructification sites and the fructified sites, they were further chosen as candidates to test potential correlations with the estimated yield level of morel ascocarps harvested from the 32 farms in this study. Indeed, the Shannon-Wiener and the inverse Simpson’s diversity indices showed positive correlations with the estimated yield level of morel ascocarps (Fig. 3B). A similar correlation was also observed between the Pielou’s evenness index and the morel yield. In comparison, the α-diversity estimators of the bacterial communities showed no correlation with the estimated yield level of morels (Fig. 3B).

The β-diversity of the fungal and bacterial communities estimated by the nonmetric multidimensional scaling (NMDS) plots showed that the community profiles were separated into two distinct groups of nonfructification versus fructification (*P* = 0.001 and 0.003 for fungal and bacterial communities, respectively) (Fig. 3C). The distinction of the fungal communities between nonfructification versus fructification (*R*^2^ = 0.035) seemed even more prominent than that of the bacterial communities (*R*^2^ = 0.027). This supported the point of view that the success or failure of morel fructification could be associated with the composition of soil microbial communities.

### Community composition.

**FIG 4:**
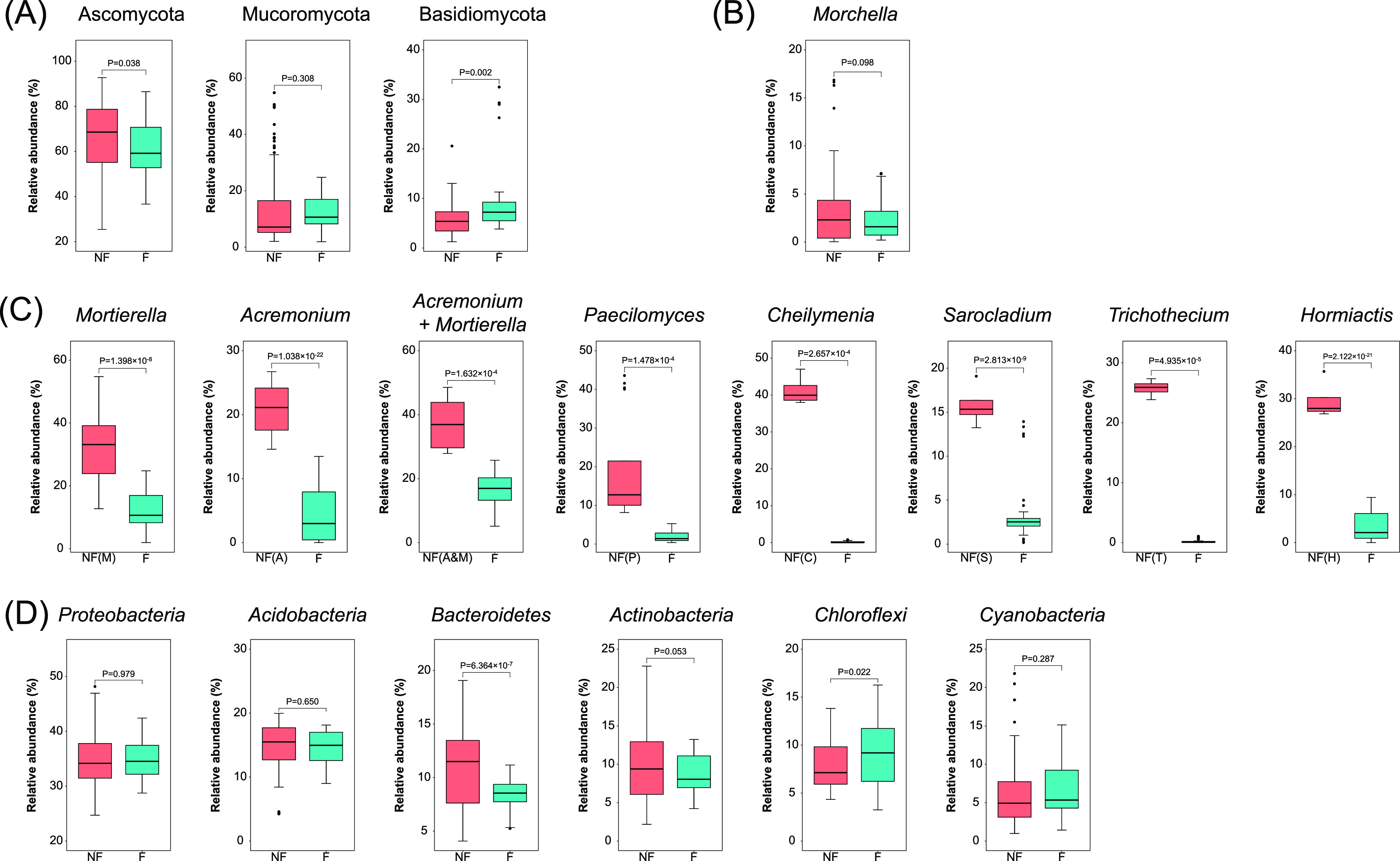
(A) Relative abundances of major phyla in the fungal communities, compared between the nonfructification sites and the fructified sites. (B) Relative abundance of the morel mycelium (*Morchella* genus) in the fungal communities. (C) Relative abundances of the predominant fungal genera, which were considered responsible for the eight patterns of nonfructification. For each nonfructification pattern, the comparison was between the nonfructification sites belonging to this pattern and the successfully fructified sites. (D) Relative abundances of major phyla in the bacterial communities. Complete bar charts showing the composition of all the fungal and bacterial phyla are provided in Fig. S1, while complete heat maps of all the fungal and bacterial genera are provided in Fig. S2.

The soil fungal communities of the morel farms were mainly composed of Ascomycota, Mucoromycota, and Basidiomycota at the phylum level (Fig. S1). The nonfructification sites had a higher proportion (*P* = 0.038) of Ascomycota and a lower proportion (*P* = 0.002) of Basidiomycota than the fructified sites, while the proportion of Mucoromycota showed no difference (*P* = 0.308) (Fig. 4A). When looking at the fungal community composition at the genus level, we found that the morel genus *Morchella* was present in all the investigated soil samples (Fig. S2). The relative abundance of *Morchella* in the fungal communities showed no significant difference between the nonfructification sites and the fructified sites (*P* > 0.05), indicating that the failure of fructification in the affected farms was not due to vanishing of the morel mycelium from the soils.

The microbial communities in most of the nonfructification sites contained a predominant genus of environmental fungi other than the morel (Fig. S2). This pattern was less typical for the bacterial communities. According to the top predominant genera in the fungal communities, eight patterns could be identified among the nonfructification sites (Fig. 4C): NF(M), predominated by *Mortierella*, containing SF03, SF05, MZ02, ZJ02, ZJ04, and JT03; NF(A), predominated by *Acremonium*, containing ZJ05, ZJ06, JT02, JT06, and ZY04; NF(A&M), predominated by two major genera of *Acremonium* and *Mortierella*, containing ZJ01 and ZY05; NF(P), predominated by *Paecilomyces*, containing JT01, JT07, XD01, and CZ01; NF(C), predominated by *Cheilymenia*, containing SF01; NF(S), predominated by *Sarocladium*, containing JT05; NF(T), predominated by *Trichothecium*, containing JT08; and NF(H), predominated by *Hormiactis*, containing JT09. Except for NF(A&M), the patterns each had a predominant genus in the fungal communities of the nonfructification sites that had a significantly higher proportion (all *P* < 0.001) than those of the fructified sites (Fig. 4C). For the NF(A&M) pattern, the *Acremonium* and *Mortierella* in the nonfructification sites was significantly more abundant (*P* < 0.001) than in the fructified sites. This was in line with the trend of α-diversity estimators, in which the fungal communities of the nonfructification sites had a greater extent of unevenness than the sites that fructified successfully.

*Proteobacteria* was the most abundant phylum among the bacterial communities, while *Acidobacteria*, *Actinobacteria*, *Bacteroidetes*, *Chloroflexi*, and *Cyanobacteria* were major phyla as well (Fig. 4D). The nonfructification sites had a higher proportion of *Bacteroidetes* (*P* < 0.001) and a lower proportion of *Chloroflexi* (*P* = 0.022), while the proportions of *Proteobacteria*, *Acidobacteria*, *Actinobacteria*, and *Cyanobacteria* were quite similar (all *P* > 0.05). No predominant taxon was observed in the bacterial communities, either in the nonfructification samples or in the fructified ones.

### Indicator fungal taxa associated with nonfructification.

**FIG 5:**
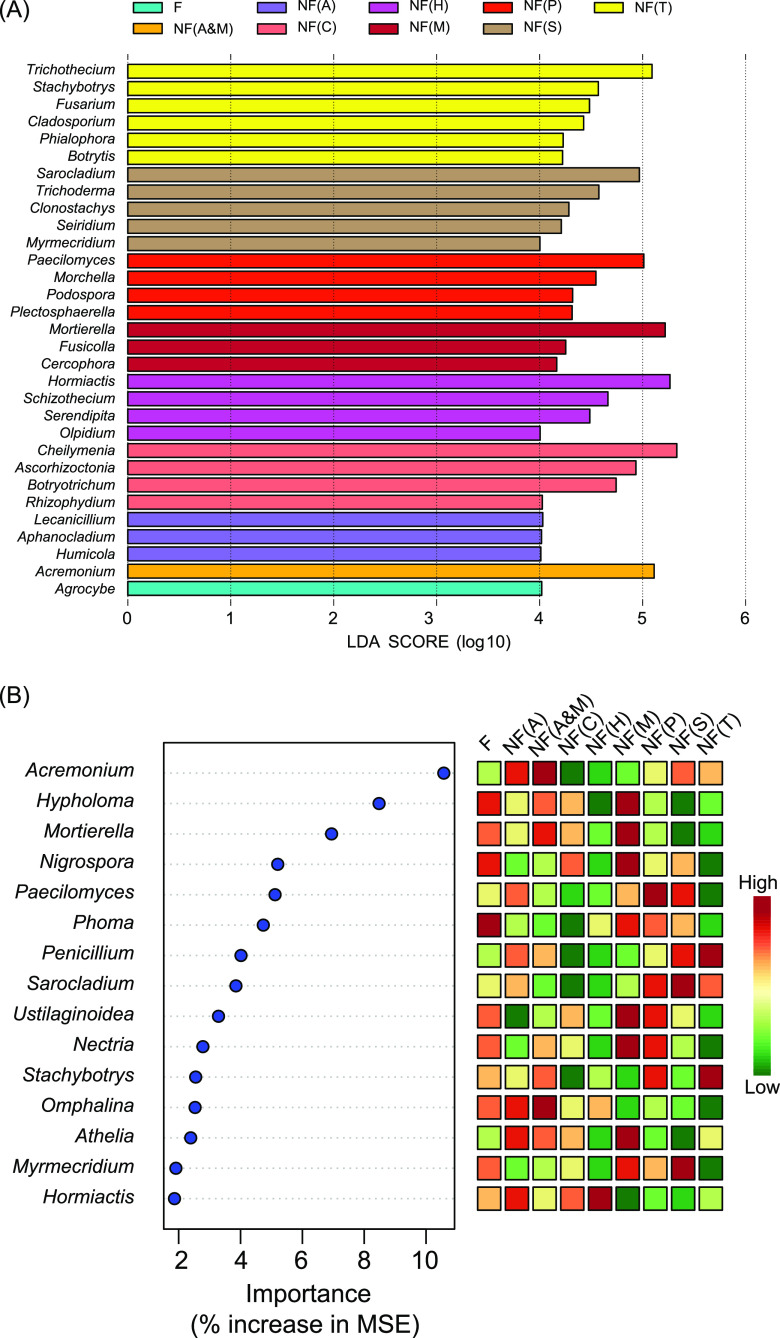
Indicator genera of the eight patterns of fungal communities associated with the nonfructification sites, predicted by the LEfSe (A) and the random-forest machine-learning method (B).

Linear discriminant analysis (LDA) effect size (LEfSe) and random-forest analyses were used to infer fungal taxa that were predictive of nonfructification. The results of the LEfSe analysis showed that the most prominent indicator fungal genera in the eight nonfructification patterns (Fig. 5A) generally corresponded to the predominant genera with the highest relative abundance (Fig. 4C), except that *Acremonium* was enriched to an even greater extent in the NF(A&M) pattern than in the NF(A) pattern. The random-forest machine-learning method was adopted to identify the major statistically significant predictors of the different patterns of community composition from the top 30 prominent fungal genera. The results revealed that *Acremonium* together with *Omphalina* was highly predictive for the patterns NF(A) and NF(A&M) (Fig. 5B). *Mortierella* together with *Hypholoma*, *Nigrospora*, *Ustilaginoidea*, *Nectria*, and *Athelia* was highly predictive of the NF(M) pattern. *Paecilomyces* was highly predictive of the NF(P) pattern. *Sarocladium* together with *Myrmecridium* was highly predictive of the NF(S) pattern. *Hormiactis* was highly predictive of the NF(P) pattern. A majority of the most predominant fungal genera in the communities (Fig. 4C) matched the results determined by the random-forest machine-learning method (Fig. 5B). Taking the results into account together, *Acremonium*, *Mortierella*, and *Paecilomyces* were selected as indicator fungal genera that are mostly associated with the microbial communities of nonfructification sites.

### Predicted trophic modes and ecological guilds of fungal communities.

**FIG 6:**
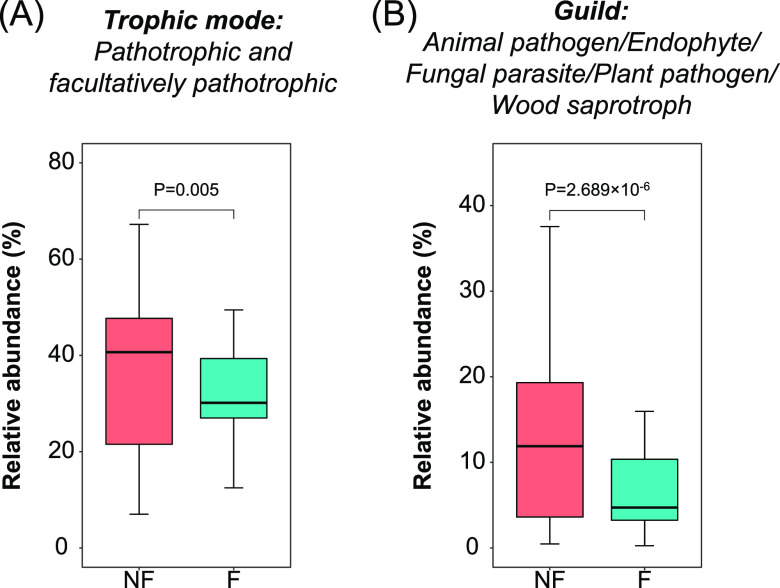
Trophic mode (A) and fungal guild (B) with significant differences (at *P* < 0.01) in their relative abundances in the soil fungal communities between the nonfructification sites and the successfully fructified sites. A bar chart showing all trophic modes is provided in Fig. S3. A heat map showing all fungal guilds is provided in Fig. S4. A complete table of the original prediction results of FUNGuild for all fungal OTUs is provided in Table S3.

A total of 8,289 fungal operational taxonomic units (OTUs) were *in silico* predicted for their trophic modes and ecological guilds by FUNGuild. The 8,289 fungal OTUs included 621 with a confidence ranking at highly probable, 3,326 at probable, and 1,516 at possible. A total of 2,112 OTUs exhibited unknown taxonomy, and 714 OTUs with known taxonomy were not assigned to any guild (Table S3). Pathotrophic and facultatively pathotrophic fungi had a significantly higher proportion in the non-fructification sites (*P* = 0.005) (Fig. 6A). Among the major guilds, “Animal pathogen/Endophyte/Fungal parasite/Plant pathogen/Wood saprotroph” (*P* < 0.001) and “Endophyte/Epiphyte/Fungal parasite/Insect parasite” had a significantly higher proportion in the nonfructification sites (*P* = 0.001) (Fig. 6B).

## DISCUSSION

This study is based on a large-scale investigation of morel farms, which analyzed soil samples during the fructification phase for the fructified sites and the expected fructification phase for the nonfructification sites. According to our criteria for site selection (see Materials and Methods), the soil type and terrain of the 21 nonproductive and the 11 productive farms were the same. The soils were all free from chemical input of killing agents. The farms were all located in a plain region with a diameter less than 200 km. They cultivated the same strain of morel obtained from the same supplier. The morels were sown at similar times. Mycelia emerging from the soil, conidiation, and sclerotium formation in the exogenous nutrient bags were observed in both the productive and the nonproductive farms at similar times. This means that the difference between fructification and nonfructification was unlikely to have been caused by vitality or other biological factors in the morel spawn. The standardized field management criteria in combination with electronic-sensor monitoring as described in Materials and Methods guaranteed that the ranges and the fluctuation patterns of the environmental factors were similar across the investigated farms. In general, unfavorable levels of soil physiochemical characteristics could be expected to inhibit mushroom fructification. However, in our case, the soil physiochemical characteristics were unlikely to have been the key factors correlated with nonfructification versus fructification (Fig. 1D). The soils were sampled at a single time point. Soil samples were not collected before fructification or at even earlier stages in this study because it was impossible to foresee which farms were going to fructify successfully and which farms would have no fructification. This could be a limitation for understanding the dynamic ecological processes of soil microbial communities linked to the success or failure of morel fructification. However, being a quite complex system, the soil microbial community usually shows resilience and stability (16–19). Given that the entire period of morel fructification lasts only for about 1 month, the composition of microbiota in the soils is not expected to shift drastically. Like other studies with soil samples collected at a single time point (20–24), which are still able to provide valid information and conclusions in soil microbial ecology, our investigation covering 128 samples from 32 sites could provide insights into the ecophysiological factors either benefiting or obstructing morel fructification.

Our study showed significant differences in the α-diversity estimators related to community evenness of the soil fungal and bacterial communities between the productive and nonproductive morel farms, although it cannot be ruled out that other factors of field environments and soil biology, including nonfungal and nonbacterial components of soil microbiota, might also be related to morel fructification and production. Pielou’s evenness index, measuring only community evenness, and the Shannon-Wiener and inverse Simpson’s indices, measuring both taxonomic richness and community evenness, all showed significant differences between the nonfructification sites and the fructified sites. In contrast, the ACE and Chao1 estimators, measuring only taxonomic richness, showed no significant difference. This means that community evenness is a key difference between the nonfructification sites and the fructified sites. Furthermore, a positive correlation was observed between Pielou’s evenness index of the soil fungal community and morel yield. To our knowledge, this is the first report of a correlation between the community evenness of soil microbiota and crop production. This confirmed our hypothesis that morel production could be related to soil microbial ecology.

The low community evenness in the nonfructification sites seemed to have been majorly contributed by a few predominant fungal taxa with high proportions in the communities, although the proportion derived from amplicon sequence data might have been biased due to involvement of PCR in this approach. Among the indicator fungal genera of the eight nonfructification patterns, nearly half (*Mortierella*, *Cheilymenia*, and *Sarocladium*) were recognized as nonpathogenic groups by FUNGuild. This means that the nonfructification in this study could have been caused by fungi commonly known as nonpathogenic. It is possible that the predominant fungal groups caused interference competition against the morel mycelium in the soils, resulting in the potential suppression of fructification. Indeed, some fungal taxa, such as *Cephalotrichum* spp., may outcompete *M. rufobrunnea* (the red-blushing morel produced in indoor trays), parasitize its mycelium, or inhibit its fructification (14). Another potential mechanism is that the predominant fungal groups with increased cell density triggered quorum sensing among their populations, which created unfavorable conditions for the morel mycelium to fructify. Further experiments creating soil fungal communities with or without a predominant population using methodologies of synthetic microbial ecology would be useful to verify the potential suppressive effects on morel fructification and elucidate the mechanisms of molecular signaling.

The indicator fungal taxa, which were considered responsible for the nonfructification, were also present in the soils with successful fructification, although in low proportion. It is expected that these taxa could proliferate to high proportions once the environmental conditions are appropriate, leading to new events of nonfructification. Moreover, it cannot be ruled out that some other fungal taxa, or nonfungal taxa, which were not identified as the main cause of nonfructification in this study, could become a predominant group in a soil microbial community once the conditions are suitable and then suppress the fructification of morels in a similar way. These possibilities would be potential ecological risks for morel agriculture. This study also revealed that the soil samples from the nonfructification sites had a higher proportion of *Bacteroidetes* and a lower proportion of *Chloroflexi* than the fructified sites (Fig. 4D). Similarly, the proportion of *Bacteroidetes* in semisynthetic substrata was also correlated with lower yields of morel ascocarps, while *Chloroflexi* was correlated with higher yield (25). This provides more hints that *Chloroflexi* might have beneficial effects on morel fructification, while *Bacteroidetes* seemed unfavorable. Synthetic microbial ecology would be a promising option to customize the composition of soil microbial community by enhancing the proportion of beneficial microorganisms.

Previous studies reported that a series of soilborne pathogens were able to infect young morel ascocarps that had fructified already (8–11). In all of these examples, the presence of a single pathogenic strain caused a symptom in the morel ascocarps, consistent with the Koch postulates. In comparison, the mechanism causing the failure of fructification in this study seemed more complicated and was influenced by the diversity and evenness of the whole microbial community in the soils (Fig. 3A and B), although the *in silico*-predicted proportions of pathotrophic and facultatively pathotrophic fungi were indeed higher in the nonfructification sites (Fig. 6A). To our knowledge, this phenomenon has not been observed in mushroom agriculture in previous studies, and it is also very rare in the agriculture of green-plant crops. A problem with the diversity of a whole microbial community, particularly a highly uneven composition of fungal taxa, cannot be cured by applying a single or a simple combination of chemical or biological agents against a definite microbial taxon. Instead, the answers to this kind of problem would rely on holistic field managements and treatments with the goal of altering the diversity of the soil microbial community. Feasible strategies might include applying biochar, organic fertilizer, and optimized tillage regimes. Owing to its porous structure and large surface area (26), biochar could provide numerous niches to shelter various microorganisms. In many cases, it could enhance the diversity of the soil microbial community, particularly in sandy soils with low SOC (27). Applying biochar in a low-rate and long-term manner is an effective way to facilitate higher microbial diversity (27). Organic fertilizer input has also been shown to be effective in improving the microbial diversity in soil environments (28, 29). Farmers often report that morel yield in fields without any crop rotation appears to decrease substantially, while crop rotation can maintain a high yield of morels. The increased soil microbial diversity due to crop rotation (30) might be an explanation.

Growing edible mushrooms was once recognized as “a conversation between bacteria and fungi” (15). Bacterial consortia in composted cultivating substrata showed great influences on the fructification of the button mushroom (Agaricus bisporus) as well as other commercial mushrooms (31), including *M. rufobrunnea* (14). In our study, the evenness and the predominant taxa of the soil fungal communities seemed critical to morel fructification, while the bacterial communities showed weaker influences than fungi. This suggests that soil fungal communities might play an important role in the ecophysiological factors driving morel fructification. The potential interactions between mushroom-forming fungi and soil filamentous fungi would be an interesting topic for study.

### Conclusion.

**FIG 7:**
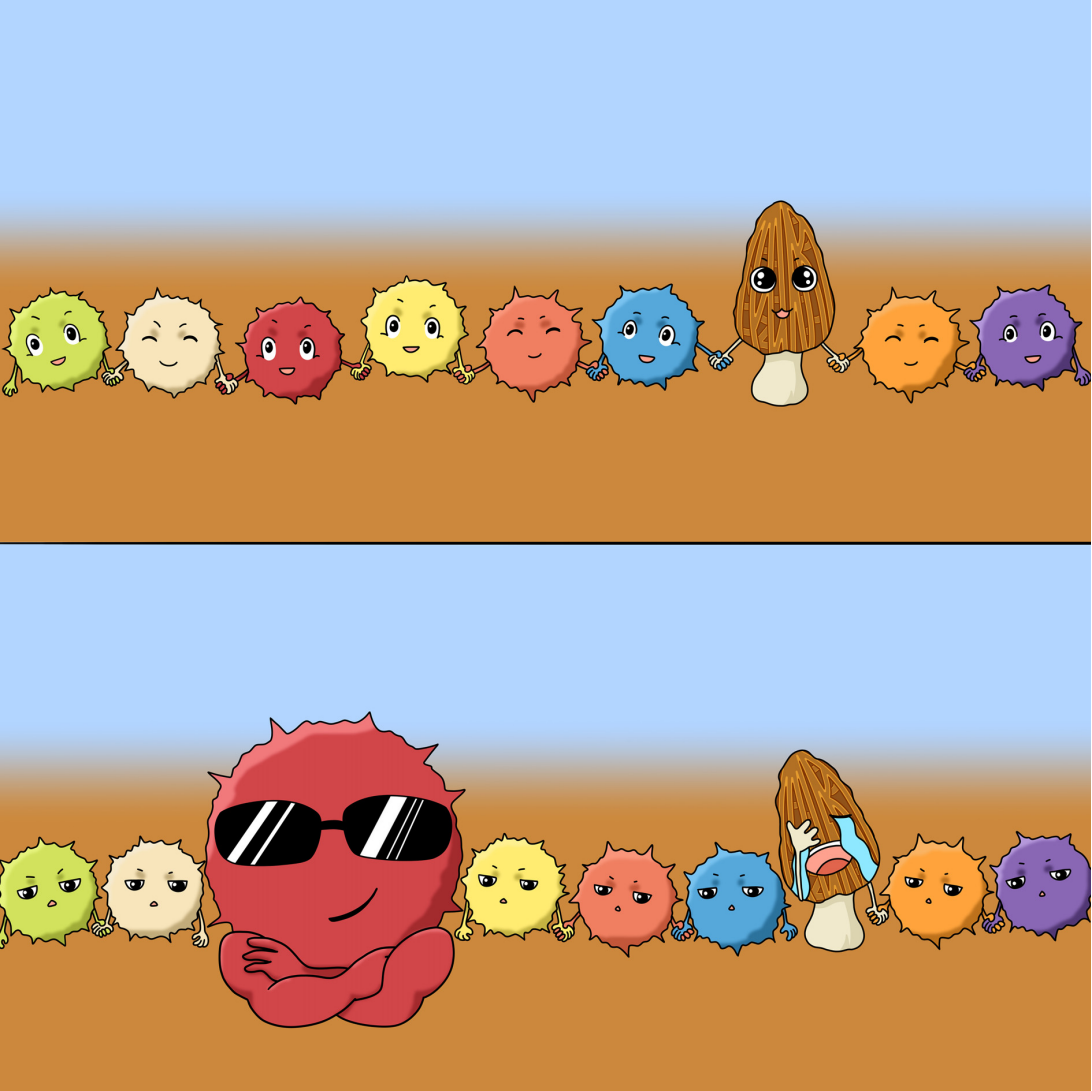
A conceptual illustration depicting the influence of community evenness of soil microbiota on large-scale morel farming.

Morel fructification in large-scale cultivation is positively correlated with the diversity and evenness of soil microbial communities. Soils with successful fructification had significantly higher diversity and evenness than those of nonfructification. The higher diversity was majorly contributed by a higher evenness of community composition rather than taxonomic richness (Fig. 7). The fungal communities of the nonfructification soils were typically dominated by one or two predominant fungal taxa. The nonfructification was not due to vanishing of the morel mycelium from the soils.

## MATERIALS AND METHODS

### Criteria for site selection.

Morel farms that fulfilled all of the following criteria were selected for analysis. (i) Their straight-line distances from the city center of the city of Chengdu, Sichuan Province, China, were less than 100 km. (ii) They were located in a plain terrain, with a soil texture of clay loam. (iii) No herbicides, fungicides, or pesticides were used prior to or during morel cultivation. (iv) They all used the same cultivated morel variety, the *M. sextelata* strain ChuanYangDuJun NO.6. (v) The inoculant for sowing (the mushroom spawn) for these farms was prepared and supplied from the same source, and all morels were sown between 28 November and 30 November 2019, using the same planting method.

The fields were all managed following the standard operating procedures for morel cultivation (32), and technicians patrolled with portable sensors to guarantee that the local environmental factors all fell into appropriate ranges (33). The appropriate illumination intensity was 2,500 to 3,500 lx when measured at noon under sunny conditions. Soil temperature was 6 to 13°C. The soil temperature reached its lowest point (6°C) every day before sunrise, while the highest point (13°C) appeared around 14:00 to 15:00. The air temperature was 6 to 22°C. The air temperature reached its lowest point (6°C) every day before sunrise, while the highest point (22°C) appeared before sunset. The ideal soil relative humidity (RH) was 20 to 23% as measured by electronic sensors. For morel cultivation, air RH should be 85 to 90% during the night. A trough appeared in the curve of air RH around 13:00 to 15:00. The lowest point of the curve trough of air RH should be above 60%. Air oxygen content was above 20.2% and air CO_2_ content was less than 600 ppm throughout the process. This monitoring ensured that the nonproduction cases that resulted from inadequate field management were excluded from the investigation.

Consequently, a total of 32 morel farms in the suburban area around Chengdu city were selected (Fig. 1B), 21 of which were nonproductive. The information on the farms is detailed in Table S1. The performance of morel fructification was reported by the farmers owning these fields. For the fields with no fructification at all, the entire area was carefully checked to ensure that no primordia formed and no ascocarp appeared. For the fields with successful fructification, the yield level of ascocarps was estimated and recorded by the farmers. The yield levels were initially reported as kilogram fresh-weight ascocarps per Mu (a Chinese conventional unit of area; 1 Mu = 667 m^2^) and then converted to kilograms per square meter.

### Soil sampling.

Soils of the investigated morel farms were sampled in early March 2020. At this time, morels in productive farms were at an early stage of development, while no morels or primordia were detected across farms with no fructification. For each field, independent of area, a cross was drawn to divide the rectangular field into four quarters with equal areas as four plots, labeled a, b, c, and d. In each plot, a three-by-three grid was drawn to divide the plot into nine equal aliquots (Fig. S5). Each aliquot was further divided by a smaller cross into four equal grids. In this way, each plot was equally divided into a grid of 36 blocks. A core of topsoil (in a cylinder shape, 5 cm in diameter and 5 cm tall) of mushroom bed was taken from a random position in every small grid. The topsoil was sampled because it was the position where the morel mycelium formed primordia and was expected to have a more significant impact on morel fructification than deeper soils. Therefore, a total of 36 soil cores were taken from each plot. The soil cores were combined and mixed thoroughly to generate a plot sample, snap-frozen with liquid nitrogen, and then kept at −80°C until they were used.

### Soil physiochemical analysis.

Soil pH, soil organic C (SOC), total N (TN), ammonium N (NH_4_^+^-N), nitrate N (NO_3_-N), available P (AP), available K (AK), exchangeable Ca (ExCa), and exchangeable Mg (ExMg) were determined. The pH was measured in a soil solution of 1:2.5 sample/water ratio as described by Borin et al. (34). To determine the SOC, the soil sample was pretreated with H_2_SO_3_ to remove inorganic carbonate as described by Aye et al. (35) and then measured by the combustion method described by Nishanth and Biswas (36). TN was determined from samples digested by H_2_SO_4_ using the micro-Kjeldahl method (37). NH_4_^+^-N was determined using the micro-Kjeldahl method procedures but without digesting the sample. NO_3_-N was determined with the salicylic acid colorimetric method described by Tian et al. (38). AP was determined with Olsen’s method (39). AK was extracted with ammonium acetate and then measured by flame atomic absorption spectrometry. ExCa and ExMg were also determined with ammonium acetate extraction followed by flame atomic absorption spectrometry as described by Behera et al. (40).

### High-throughput sequencing of community amplicons.

Soil total DNA was extracted using a cetyltrimethylammonium bromide (CTAB) extraction method (41). The solutions for the DNA extraction were freshly prepared, autoclaved, and kept sterile until use. Autoclaved pure water and quartz particles were used as two types of blank extraction controls. The blank extraction controls and the formal samples were extracted in the same batch using the same solutions as the CTAB extraction method to guarantee no microbiome contamination in the extraction reagents, as recommended by Hornung et al. (42) and Zinger et al. (43). The primers 515F (5′-GTGCCAGCMGCCGCGG-3′) and 907R (5′-CCGTCAATTCMTTTRAGTTT-3′) (44) were used to amplify the V4-V5 region of the bacterial 16S rRNA gene. The primers ITS1-F (5′-CTTGGTCATTTAGAGGAAGTAA-3′) and ITS2-R (5′-GCTGCGTTCTTCATCGATGC-3′) (45) were used to amplify the internal transcribed spacer (ITS) region of fungi. The obtained amplicons were connected to index barcodes using the NextUltra DNA library prep kit for Illumina (New England BioLabs [NEB], USA) following the manufacturer’s instructions. The samples of the blank extraction controls obtained no detectable yield of DNA extracts and generated no sequencing library of metabarcoding amplicons by the PCR amplification. This confirmed that the DNA extraction and library construction steps introduced no contamination from the exogenous microbiome.

The libraries were sequenced on an Illumina MiSeq PE250 platform at the sequencing facility of Biozeron Biological Technology Co., Ltd. (Shanghai, China), according to standard Illumina protocols. Paired-end reads were quality controlled, merged by overlapping, and analyzed with the QIIME pipeline (46). Chimeras were removed by the USEARCH tool using the UCHIME algorithm (47). Chimeras of 16S amplicons were removed with a combination of the *de novo* and the reference methods against the Gold database (http://drive5.com/uchime/uchime_download.html). Chimeras of ITS amplicons were removed with the *de novo* method.

### Bioinformatic analysis of microbial community.

OTUs of bacteria and fungi were clustered with a similarity threshold of 97% using the UPARSE algorithm (48). Bacterial OTUs were clustered against the SILVA reference database (release 132), while fungal OTUs were clustered against the UNITE v7.2 (Full UNITE+INSD data sets) as described previously (5). The OTUs were assigned to taxonomy using the RDP classifier of the QIIME pipeline. Sequencing scales were normalized among different samples. The number of assembled sequences in each sample that could be mapped to the OTU table was counted. The sample with the lowest number of sequences mapping to the OTU table was chosen as a subsampling criterion. The sequences of the other samples were all rarefied toward the subsampling criterion. Finally, the 16S amplicons of each sample were normalized to 26,612 sequences, while the ITS amplicons were normalized to 27,972. The OTU tables of the fungal and bacterial communities are provided in the supplemental material (Table S4). The coverage of OTUs in the microbial community was estimated by the rarefaction curve of OTUs (Fig. S6) as well as Good’s coverage index (Table S5). The formula used to calculate Good’s coverage index is *C *= 1 − (*n*_1_/*N*), in which *n*_1_ means the number of OTUs with only one sequence and *N* means the total number of sequences in the sample, as described at http://mothur.org/wiki/coverage/. The fungal communities had an average level of Good’s coverage index at 0.992 ± 0.003, while that of the bacterial communities was 0.935 ± 0.013, indicating that a majority of the communities had been covered by the sampling. OTUs with only one sequence (singleton OTUs) were removed prior to further statistical analysis.

The taxonomic richness of bacterial and fungal taxa was estimated by the ACE and Chao1 indices. Community diversity was estimated by the Shannon-Wiener and inverse Simpson’s indices, as used previously (5). Community evenness was estimated by Pielou’s evenness index using a calculation formula of *J* = *H*/ln*S* (49), in which *J* is Pielou’s evenness, *H* is the Shannon-Wiener index, and *S* is the number of observed OTUs. The correlation between α-diversity estimators and morel yield was analyzed using the “stat_cor” command of ggpubr. The results were visualized by the ggplot2 package. The β-diversity of the communities of different samples was estimated by NMDS analysis using Bray-Curtis distance. The OTU table was normalized using cumulative sum scaling before calculating the Bray-Curtis distance matrix, as described by Longley et al. (14). The dispersion of community groups was estimated by permutational multivariate analysis of variance (PERMANOVA), using the “adonis” command in the R package vegan (50). The significance of community dispersion was judged by *P* value of <0.05 of PERMANOVA. The relationship between microbial community profiles and soil physiochemical characteristics was determined by RDA, with the Envfit test (50) used to estimate the contribution of soil physiochemical characteristics to microbial community profiles. Differential distribution of fungal genera among the samples was determined by linear discriminant analysis (LDA) effect size (LEfSe). Significantly enriched indicator genera were judged with an LDA score (log_10_) of >4.0 and *P* value of <0.05. A classification random-forest machine-learning analysis (51) was conducted as previously used by Liu et al. (52) to identify the major statistically significant predictors of fungal genera in the microbial community composition. The analysis used the randomForest and rfPermute packages of R, as described by Wang et al. (53) and Liu et al. (52). The importance of the identified predictors was shown as the increase in the mean squared error (MSE) of prediction as described by Liu et al. (52).

### *In silico* prediction of trophic modes and ecological guilds in fungal community.

The trophic modes and ecological guilds of the fungal communities were predicted with FUNGuild (54) using the Guilds_v1.1.py at https://github.com/UMNFuN/FUNGuild. The most abundant fungal genera in the eight nonfructification patterns were manually queried with the online tool of FUNGuild at http://www.funguild.org/query.php?qText=&qDB=funguild_db&qField=taxon.

### Statistical analysis.

Significance of difference among three or more groups of samples was judged by one-way ANOVA at a *P* value of <0.05, with Tukey honestly significant difference (HSD) used for false-discovery rate correction in multiple testing. The significance of difference between two groups was judged by the *t* test at a *P* value of <0.05. PASW Statistics version 18 (IBM SPSS Inc., USA) software was used for the statistical tests.

### Data availability.

The metabarcoding data sets of bacterial 16S ribosomal DNA (rDNA) V4-V5 and fungal ITS amplicons are accessible under NCBI BioProject PRJNA713847.
