# Supplementary material for: Morel Production Related to Soil Microbial Diversity and Evenness
Source: Microbiol Spectr. 2021 Oct 13;9(2):e00229-21. doi: 10.1128/Spectrum.00229-21 (PMC8515941; doi:10.1128/Spectrum.00229-21)
Supplement: SUPPLEMENTAL FILE 1 — Supplemental material. Download SPECTRUM00229-21_Supp_1_seq1.pdf, PDF file, 2.0 MB [file spectrum00229-21_supp_1_seq1.pdf]

Online Supplemental Materials

Table S1 Information of the investigated morel farms

Sample name	Site	Latitude	Longitude	Altitude (m)	Total area (ha)	Soil texture	Estimated yield (kg m^{-2})
SF01	Libing Village, Luoshui Town, Shifang City	31.25°N	104.03°E	668	4.00	Sandy loam	0
SF02	Libing Village, Luoshui Town, Shifang City	31.25°N	104.03°E	668	0.01	Sandy loam	0.60
SF03	Yu'an Village, Luoshui Town, Shifang City	31.23°N	104.03°E	650	0.20	Sandy loam	0
SF04	Wu'yi Village, Jiandi Town, Shifang City	31.22°N	104.04°E	634	0.09	Sandy loam	0.30
SF05	Wu'yi Village, Jiandi Town, Shifang City	31.22°N	104.04°E	634	0.53	Sandy loam	0
MZ01	Wenyong Village, Fuxing Town, Mianzhu City	31.37°N	104.29°E	582	7.00	Sandy loam	0.45
MZ02	Linqiao Village, Xinglong Town, Mianzhu City	31.40°N	104.24°E	613	2.54	Sandy loam	0
MZ03	Linqiao Village, Xinglong Town, Mianzhu City	31.40°N	104.24°E	613	0.80	Sandy loam	0.15
ZJ01	Guanghui Village, Nanhua Town, Zhongjiang County, Chengdu City	31.02°N	104.64°E	428	4.67	Sandy loam	0
ZJ02	Guanghui Village, Nanhua Town, Zhongjiang County, Chengdu City	31.02°N	104.64°E	428	3.34	Sandy loam	0
ZJ03	Diaolougou Village, Huilong Town, Zhongjiang County, Chengdu City	31.03°N	104.74°E	420	17.54	Sandy loam	0.45
ZJ04	Beita Village, Dongbei Town, Zhongjiang County, Chengdu City	31.06°N	104.68°E	435	4.67	Sandy loam	0
ZJ05	Suba Village, Nanfang Town, Zhongjiang County, Chengdu City	31.09°N	104.65°E	435	20.68	Sandy loam	0
ZJ06	Jinma Village, Muxing Town, Zhongjiang County, Chengdu City	31.12°N	104.65°E	442	19.34	Sandy loam	0
JT01	Guanyin Village, Zhuhao Town, Jintang County, Chengdu City	30.64°N	104.74°E	430	36.02	Clay loam	0
JT02	Zhuhaosi Community, Zhuhao Town, Jintang County, Chengdu City	30.64°N	104.73°E	425	13.34	Clay loam	0
JT03	Bakesong Village, Zhuhao Town, Jintang County, Chengdu City	30.61°N	104.71°E	445	12.00	Clay loam	0
JT04	Bakesong Village, Zhuhao Town, Jintang County, Chengdu City	30.62°N	104.71°E	449	2.67	Clay loam	0.30
JT05	Bakesong Village, Zhuhao Town, Jintang County, Chengdu City	30.62°N	104.71°E	446	5.34	Clay loam	0
JT06	Honglian Village, Zhaojia Town, Jintang County, Chengdu City	30.82°N	104.60°E	457	1.20	Clay loam	0
JT07	Sanhebei Village, Fuxing Town, Jintang County, Chengdu city	30.85°N	104.60°E	467	0.40	Clay loam	0
JT08	Yanghe Community, Zhaojia Town, Jintang County, Chengdu City	30.82°N	104.58°E	464	1.33	Clay loam	0
JT09	Yanghe Community, Zhaojia Town, Jintang County, Chengdu City	30.82°N	104.58°E	464	4.00	Clay loam	0
XD01	Taixing Town, Xindu District, Chengdu City	30.78°N	104.22°E	480	0.03	Clay loam	0
XD02	Taixing Town, Xindu District, Chengdu City	30.78°N	104.22°E	480	0.03	Clay loam	0.45
XD03	Taixing Town, Xindu District, Chengdu City	30.78°N	104.22°E	480	0.04	Clay loam	0.30
ZY01	Erwan Village, Xiangfu Town, Yanjiang District, Ziyang City	30.18°N	104.49°E	405	0.67	Clay loam	0.38
ZY02	Erwan Village, Xiangfu Town, Yanjiang District, Ziyang City	30.18°N	104.49°E	405	2.00	Clay loam	0.38
ZY03	Erwan Village, Xiangfu Town, Yanjiang District, Ziyang City	30.18°N	104.49°E	405	1.33	Clay loam	0.38
ZY04	Erwan Village, Xiangfu Town, Yanjiang District, Ziyang City	30.18°N	104.49°E	405	0.07	Clay loam	0
ZY05	Erwan Village, Xiangfu Town, Yanjiang District, Ziyang City	30.18°N	104.49°E	405	0.07	Clay loam	0
CZ01	Erjiang Village, Huaiyuan Town, Chongzhou City	30.75°N	103.60°E	580	16.68	Sandy loam	0

Table S2. P-values of ANOVA comparison of soil physiochemical characteristics among the eight zones in Fig. 2A.

P-value		pH	SOC	TN	NH_4^+-N	NO_3^--N	AP	AK	ExCa	ExMg
Zone1	Zone2	0.153	0.097	0.998	0.983	1.000	0.000	0.809	0.614	0.010
	Zone3	0.606	0.006	0.996	1.000	1.000	0.000	0.777	0.410	0.043
	Zone4	0.968	0.024	0.998	0.316	1.000	0.000	0.000	0.639	0.006
	Zone5	0.816	0.275	0.999	1.000	0.952	0.000	1.000	0.901	0.116
	Zone6	0.022	0.000	0.021	0.004	0.102	0.000	0.166	0.104	0.145
	Zone7	0.000	0.000	0.371	0.455	0.330	0.000	0.744	1.000	0.043
	Zone8	0.000	0.000	0.000	0.000	0.000	0.000	1.000	1.000	0.877
Zone2	Zone1	0.153	0.097	0.998	0.983	1.000	0.000	0.809	0.614	0.010
	Zone3	0.941	1.000	1.000	0.993	1.000	0.685	1.000	1.000	0.963
	Zone4	0.659	1.000	1.000	0.967	1.000	0.929	0.216	1.000	1.000
	Zone5	0.005	0.998	0.945	0.970	0.957	0.162	0.961	0.999	0.966
	Zone6	0.998	0.013	0.225	0.150	0.246	0.155	0.975	0.987	0.988
	Zone7	0.175	0.026	0.196	0.132	0.443	0.026	1.000	0.802	0.985
	Zone8	0.000	0.000	0.000	0.000	0.000	0.000	0.911	0.997	0.011
Zone3	Zone1	0.606	0.006	0.996	1.000	1.000	0.000	0.777	0.410	0.043
	Zone2	0.941	1.000	1.000	0.993	1.000	0.685	1.000	1.000	0.963
	Zone4	0.995	1.000	1.000	0.365	1.000	0.017	0.032	1.000	0.993
	Zone5	0.031	0.954	0.894	1.000	0.922	0.923	0.968	0.998	1.000
	Zone6	0.543	0.006	0.087	0.004	0.095	0.883	0.875	0.960	1.000
	Zone7	0.000	0.010	0.064	0.290	0.241	0.484	1.000	0.654	1.000
	Zone8	0.000	0.000	0.000	0.000	0.000	0.000	0.929	0.996	0.044
Zone4	Zone1	0.968	0.024	0.998	0.316	1.000	0.000	0.000	0.639	0.006
	Zone2	0.659	1.000	1.000	0.967	1.000	0.929	0.216	1.000	1.000
	Zone3	0.995	1.000	1.000	0.365	1.000	0.017	0.032	1.000	0.993
	Zone5	0.225	0.993	0.927	0.304	0.986	0.001	0.004	1.000	0.993
	Zone6	0.218	0.004	0.097	0.542	0.061	0.002	0.868	0.910	0.999
	Zone7	0.000	0.006	0.101	0.001	0.476	0.000	0.067	0.844	0.998
	Zone8	0.000	0.000	0.000	0.000	0.000	0.000	0.066	0.999	0.014
Zone5	Zone1	0.816	0.275	0.999	1.000	0.952	0.000	1.000	0.901	0.116
	Zone2	0.005	0.998	0.945	0.970	0.957	0.162	0.961	0.999	0.966
	Zone3	0.031	0.954	0.894	1.000	0.922	0.923	0.968	0.998	1.000
	Zone4	0.225	0.993	0.927	0.304	0.986	0.001	0.004	1.000	0.993
	Zone6	0.000	0.001	0.006	0.005	0.008	1.000	0.401	0.781	1.000
	Zone7	0.000	0.001	0.835	0.636	0.973	0.998	0.953	0.979	1.000
	Zone8	0.000	0.000	0.000	0.000	0.000	0.000	0.999	1.000	0.067
Zone6	Zone1	0.022	0.000	0.021	0.004	0.102	0.000	0.166	0.104	0.145
	Zone2	0.998	0.013	0.225	0.150	0.246	0.155	0.975	0.987	0.988
	Zone3	0.543	0.006	0.087	0.004	0.095	0.883	0.875	0.960	1.000
	Zone4	0.218	0.004	0.097	0.542	0.061	0.002	0.868	0.910	0.999
	Zone6	0.000	0.001	0.006	0.005	0.008	1.000	0.401	0.781	1.000
	Zone7	0.590	0.998	0.000	0.000	0.000	1.000	0.929	0.206	1.000
	Zone8	0.000	0.000	0.000	0.000	0.000	0.000	0.514	0.886	0.069
Zone7	Zone1	0.000	0.000	0.371	0.455	0.330	0.000	0.744	1.000	0.043
	Zone2	0.175	0.026	0.196	0.132	0.443	0.026	1.000	0.802	0.985
	Zone3	0.000	0.010	0.064	0.290	0.241	0.484	1.000	0.654	1.000
	Zone4	0.000	0.006	0.101	0.001	0.476	0.000	0.067	0.844	0.998
	Zone5	0.000	0.001	0.835	0.636	0.973	0.998	0.953	0.979	1.000
	Zone6	0.590	0.998	0.000	0.000	0.000	1.000	0.929	0.206	1.000

	**Zone8**	0.000	0.000	0.000	0.000	0.000	0.000	0.911	1.000	0.040
**Zone8**	**Zone1**	0.000	0.000	0.000	0.000	0.000	0.000	1.000	1.000	0.877
	**Zone2**	0.000	0.000	0.000	0.000	0.000	0.000	0.911	0.997	0.011
	**Zone3**	0.000	0.000	0.000	0.000	0.000	0.000	0.929	0.996	0.044
	**Zone4**	0.000	0.000	0.000	0.000	0.000	0.000	0.066	0.999	0.014
	**Zone5**	0.000	0.000	0.000	0.000	0.000	0.000	0.999	1.000	0.067
	**Zone6**	0.000	0.000	0.000	0.000	0.000	0.000	0.514	0.886	0.069
	**Zone7**	0.000	0.000	0.000	0.000	0.000	0.000	0.911	1.000	0.040

Table S3. Original prediction results of FUNGuild for all fungal OTUs. The table is of big size and is therefore provided as an individual Excel file available online: TableS3.xlsx.

Table S4. OTU tables of the fungal and bacterial communities. The table is of big size and is therefore provided as multiple working-sheets in an individual Excel file available online: TableS4.xlsx.

Table S5. α-diversity and the Good's Coverage Index of the fungal and bacterial communities.

Samples	Fungal OTUs						Bacterial OTUs					
	Good's Coverage	ACE	Chao1	Shannon-Wiener	Inverse Simpson	Evenness	Good's Coverage	ACE	Chao1	Shannon-Wiener	Inverse Simpson	Evenness
**SF01a**	0.992	1181	1158	3.052	5.144	0.487	0.927	11544	11351	7.654	278.087	0.867
**SF01b**	0.994	1533	1673	3.184	5.923	0.494	0.939	11622	11338	7.499	154.847	0.844
**SF01c**	0.992	1430	1408	3.160	5.659	0.436	0.910	13302	10908	7.466	170.999	0.840
**SF01d**	0.990	1224	1185	2.805	4.191	0.390	0.938	11765	11488	7.649	258.465	0.855
**SF03a**	0.988	1716	1713	4.508	10.601	0.594	0.936	11215	10992	7.330	88.098	0.809
**SF03b**	0.989	1561	1511	4.496	10.906	0.596	0.921	9836	9452	6.940	42.957	0.795
**SF03c**	0.990	1646	1609	4.732	14.169	0.609	0.938	11334	10916	7.131	53.220	0.784
**SF03d**	0.991	1662	1644	4.639	14.238	0.599	0.922	10225	9817	7.229	83.278	0.839
**SF05a**	0.994	1460	1446	4.199	8.750	0.567	0.937	11299	11009	7.888	904.159	0.896
**SF05b**	0.991	1345	1385	4.226	9.320	0.542	0.935	11459	11298	7.889	926.784	0.883
**SF05c**	0.990	1379	1346	3.999	7.174	0.498	0.918	13307	11183	7.870	784.314	0.889
**SF05d**	0.990	1538	1343	3.687	6.898	0.497	0.921	10571	10326	7.851	1044.932	0.892
**MZ02a**	0.997	1258	1288	5.272	56.313	0.704	0.947	11339	10950	7.986	1199.041	0.891
**MZ02b**	0.996	1396	1428	5.269	56.287	0.676	0.942	10633	10472	7.909	1083.424	0.887
**MZ02c**	0.995	1236	1261	5.297	58.692	0.726	0.913	9664	9461	7.890	1168.224	0.904
**MZ02d**	0.996	1138	1185	5.157	54.597	0.697	0.916	10099	9877	7.916	1203.369	0.907
**ZJ01a**	0.987	1568	1539	4.428	17.202	0.609	0.921	11640	9410	7.549	594.884	0.873
**ZJ01b**	0.986	1717	1730	4.325	14.467	0.602	0.947	11062	10689	7.580	527.148	0.846
**ZJ01c**	0.990	2017	1960	4.006	9.492	0.560	0.917	11062	9039	7.456	414.938	0.862
**ZJ01d**	0.992	1838	1809	4.407	14.486	0.592	0.948	10974	10578	7.586	528.821	0.837
**ZJ02a**	0.991	1569	1597	4.398	29.556	0.572	0.931	10209	9940	7.605	480.538	0.866
**ZJ02b**	0.988	1483	1485	4.378	29.424	0.566	0.929	10712	10514	7.631	504.796	0.840
**ZJ02c**	0.993	1644	1653	4.236	24.968	0.551	0.949	10870	10555	7.458	274.424	0.827
**ZJ02d**	0.992	1552	1520	4.313	27.611	0.574	0.940	10561	10221	7.649	523.013	0.857
**ZJ04a**	0.992	1830	1808	4.528	16.359	0.609	0.946	10415	10188	7.690	404.204	0.869
**ZJ04b**	0.992	1830	1785	4.585	16.086	0.572	0.948	10600	10331	7.694	322.165	0.865

**ZJ04c**	0.991	1844	1890	4.585	17.568	0.571	0.947	10181	9889	7.709	357.526	0.873
**ZJ04d**	0.988	1496	1527	4.459	16.143	0.565	0.946	10280	9969	7.684	378.358	0.871
**ZJ05a**	0.993	1621	1602	4.736	24.920	0.574	0.946	9624	9483	7.533	465.116	0.842
**ZJ05b**	0.990	1570	1540	4.849	30.446	0.634	0.948	9721	9503	7.567	497.265	0.861
**ZJ05c**	0.988	1643	1629	4.747	26.121	0.656	0.946	9430	9254	7.366	287.522	0.825
**ZJ05d**	0.989	1610	1633	4.700	28.329	0.648	0.954	9826	9754	7.590	568.505	0.845
**ZJ06a**	0.992	1355	1294	4.019	17.650	0.670	0.950	6738	6500	6.932	163.988	0.831
**ZJ06b**	0.994	1364	1370	4.051	17.304	0.571	0.953	6843	6733	6.992	191.132	0.830
**ZJ06c**	0.994	1505	1540	4.019	16.278	0.559	0.953	7344	7144	7.127	224.467	0.827
**ZJ06d**	0.994	1393	1400	3.921	15.712	0.581	0.951	8289	6942	6.996	188.430	0.822
**JT01a**	0.987	1673	1665	4.835	40.043	0.665	0.923	9747	9741	7.814	921.659	0.897
**JT01b**	0.988	1702	1659	4.931	48.400	0.661	0.913	11981	9876	7.832	952.381	0.887
**JT01c**	0.990	1831	1790	5.007	51.568	0.685	0.941	11532	11228	7.861	838.223	0.878
**JT01d**	0.987	1600	1541	4.965	49.495	0.665	0.917	11396	9473	7.809	930.233	0.894
**JT02a**	0.990	1274	1280	4.037	12.007	0.587	0.954	9954	9848	7.412	539.084	0.826
**JT02b**	0.993	1717	1731	4.336	15.042	0.577	0.932	10671	8788	7.365	544.662	0.849
**JT02c**	0.988	1479	1500	4.211	13.113	0.594	0.940	11080	9417	7.378	498.753	0.827
**JT02d**	0.992	1731	1689	4.154	13.004	0.554	0.923	10316	8525	7.381	512.033	0.838
**JT03a**	0.993	1768	1777	4.614	12.808	0.608	0.921	11167	10846	7.952	963.391	0.892
**JT03b**	0.989	1689	1676	4.686	15.271	0.623	0.928	11708	11399	7.917	784.929	0.898
**JT03c**	0.994	1895	1867	4.849	16.518	0.612	0.934	12158	11853	7.987	947.867	0.893
**JT03d**	0.993	1970	2000	4.624	14.536	0.585	0.894	13130	10354	7.863	860.585	0.904
**JT05a**	0.991	1827	1771	4.282	18.225	0.563	0.925	10958	8930	7.441	519.751	0.846
**JT05b**	0.991	1906	1888	4.723	29.362	0.664	0.946	11050	10842	7.541	518.672	0.835
**JT05c**	0.992	2048	1996	4.737	29.452	0.603	0.938	10227	9957	7.497	514.403	0.843
**JT05d**	0.991	2034	2058	4.846	35.909	0.625	0.948	10810	10620	7.566	567.859	0.837
**JT06a**	0.993	1759	1758	4.840	27.236	0.654	0.941	8240	8064	7.303	406.174	0.827
**JT06b**	0.992	1726	1703	4.832	29.053	0.664	0.947	8693	8465	7.311	427.168	0.824
**JT06c**	0.991	1646	1627	4.883	29.433	0.651	0.950	9548	9270	7.236	362.713	0.820
**JT06d**	0.989	1618	1642	4.978	40.337	0.692	0.947	8752	8513	7.291	400.160	0.826
**JT07a**	0.987	1711	1677	5.040	51.714	0.691	0.928	13151	11077	7.787	928.505	0.873
**JT07b**	0.991	1929	1918	5.339	72.406	0.702	0.921	9866	9469	7.683	653.595	0.870
**JT07c**	0.991	2065	2012	5.313	69.517	0.681	0.905	12084	9847	7.725	799.361	0.882
**JT07d**	0.992	2018	1964	5.344	71.271	0.692	0.931	11660	11412	7.801	823.045	0.857
**JT08a**	0.995	1289	1254	3.875	13.544	0.578	0.958	9401	9197	7.687	853.971	0.866
**JT08b**	0.993	1163	1125	3.779	11.110	0.553	0.952	9128	9051	7.678	838.223	0.869
**JT08c**	0.994	1197	1159	3.837	12.388	0.556	0.957	9245	9242	7.678	825.083	0.860
**JT08d**	0.995	1281	1270	3.805	11.784	0.573	0.927	7976	7922	7.618	835.422	0.895
**JT09a**	0.994	1227	1213	3.908	10.838	0.532	0.920	10191	8171	7.480	654.450	0.870
**JT09b**	0.995	1300	1312	3.885	11.274	0.597	0.948	9919	9782	7.514	563.380	0.846
**JT09c**	0.995	1220	1236	3.829	10.112	0.516	0.956	9379	9146	7.446	501.756	0.840
**JT09d**	0.994	1218	1184	3.813	8.931	0.535	0.947	9622	9413	7.432	492.611	0.848
**XD01a**	0.992	1556	1533	4.390	20.234	0.604	0.948	10679	10666	7.687	783.699	0.853
**XD01b**	0.991	1502	1478	4.398	20.926	0.601	0.950	10719	10526	7.643	738.552	0.850
**XD01c**	0.993	1574	1559	4.349	19.734	0.552	0.933	9700	9388	7.599	713.776	0.869
**XD01d**	0.990	1586	1570	4.402	20.714	0.599	0.950	10614	10457	7.643	733.676	0.859
**ZY04a**	0.993	1412	1385	4.518	25.094	0.572	0.954	10031	9775	7.713	721.501	0.855
**ZY04b**	0.994	1309	1289	4.314	17.301	0.571	0.927	9293	9082	7.665	725.689	0.874
**ZY04c**	0.993	1477	1493	4.493	19.878	0.565	0.944	9994	9816	7.673	705.219	0.859
**ZY04d**	0.995	1391	1399	4.320	16.698	0.574	0.947	9579	9320	7.636	665.779	0.867
**ZY05a**	0.990	1850	1844	4.995	33.376	0.634	0.915	9340	9083	7.767	931.966	0.882

**ZY05b**	0.992	1797	1811	5.028	35.003	0.656	0.940	10057	9820	7.777	922.509	0.872
**ZY05c**	0.987	1733	1757	4.991	39.717	0.648	0.911	11229	9353	7.755	934.579	0.883
**ZY05d**	0.993	1749	1701	4.946	33.364	0.670	0.946	10668	10650	7.799	895.255	0.869
**CZ01a**	0.992	1552	1278	3.594	7.297	0.524	0.941	8649	8429	7.424	481.696	0.843
**CZ01b**	0.992	1425	1151	3.514	6.743	0.553	0.932	8863	8688	7.437	482.160	0.863
**CZ01c**	0.994	1337	1346	3.699	7.919	0.486	0.931	8601	8323	7.407	453.721	0.854
**CZ01d**	0.994	1235	1204	3.661	7.886	0.496	0.918	9268	7382	7.379	527.983	0.865
**SF02a**	0.993	1696	1719	5.224	69.517	0.689	0.925	12080	9934	7.568	422.297	0.863
**SF02b**	0.987	1658	1642	5.284	75.030	0.730	0.937	10409	10216	7.430	282.247	0.843
**SF02c**	0.992	1700	1685	5.292	79.214	0.708	0.915	11203	9156	7.434	299.133	0.845
**SF02d**	0.993	1757	1742	5.287	79.777	0.695	0.945	11237	11043	7.488	222.866	0.836
**SF04a**	0.990	1925	1929	4.785	34.092	0.654	0.923	12440	10169	7.534	438.982	0.844
**SF04b**	0.987	1824	1760	4.770	35.217	0.687	0.913	11750	9265	7.406	341.530	0.837
**SF04c**	0.991	1794	1797	4.509	23.785	0.633	0.912	10852	8629	7.210	246.427	0.840
**SF04d**	0.983	1855	1846	4.877	38.319	0.669	0.921	12442	10225	7.556	451.671	0.858
**MZ01a**	0.998	792	801	4.693	38.239	0.690	0.936	9341	9031	7.787	956.938	0.883
**MZ01b**	0.997	708	711	4.545	33.290	0.690	0.940	9546	9456	7.869	1109.878	0.888
**MZ01c**	0.997	699	699	4.571	32.290	0.712	0.952	10351	10223	7.932	1136.364	0.886
**MZ01d**	0.997	724	757	4.660	36.992	0.710	0.950	9765	9600	7.838	1018.330	0.882
**MZ03a**	0.996	1026	1046	4.723	33.569	0.686	0.923	10382	10059	7.788	668.896	0.877
**MZ03b**	0.998	596	603	4.573	38.123	0.703	0.935	10369	10151	7.784	584.795	0.878
**MZ03c**	0.997	1125	1129	5.359	72.897	0.724	0.946	11381	11170	7.831	622.665	0.904
**MZ03d**	0.997	1252	1247	5.394	64.533	0.746	0.908	9744	9673	7.764	611.247	0.883
**ZJ03a**	0.993	1759	1764	5.047	59.151	0.678	0.935	9041	8695	7.592	726.216	0.880
**ZJ03b**	0.991	1778	1751	4.973	54.145	0.683	0.948	9974	9691	7.651	770.416	0.868
**ZJ03c**	0.993	1765	1744	5.022	54.564	0.689	0.932	8881	8628	7.609	781.250	0.871
**ZJ03d**	0.992	1693	1699	5.000	59.323	0.689	0.946	9343	8967	7.597	714.286	0.877
**JT04a**	0.986	1866	1863	4.901	45.415	0.651	0.947	10890	10663	7.711	758.725	0.859
**JT04b**	0.986	1866	1863	4.901	45.415	0.655	0.935	10321	10130	7.685	727.273	0.909
**JT04c**	0.988	1848	1861	4.851	44.385	0.648	0.947	11001	10744	7.722	730.994	0.849
**JT04d**	0.991	1896	1835	4.791	40.497	0.629	0.917	11038	8961	7.626	741.290	0.875
**XD02a**	0.993	1751	1775	5.443	91.258	0.722	0.957	9060	8884	7.386	366.703	0.825
**XD02b**	0.990	1577	1620	5.438	94.598	0.731	0.926	9125	7650	7.261	357.398	0.829
**XD02c**	0.993	1723	1713	5.415	86.873	0.726	0.934	9808	8175	7.291	347.705	0.852
**XD02d**	0.993	1609	1599	5.439	87.017	0.758	0.926	9709	8032	7.217	205.508	0.822
**XD03a**	0.993	1635	1616	4.734	26.909	0.650	0.943	11027	10644	7.936	1078.749	0.883
**XD03b**	0.993	1602	1582	4.749	25.679	0.664	0.943	11368	11046	7.996	1129.944	0.885
**XD03c**	0.992	1516	1527	4.831	32.445	0.689	0.927	10361	10199	7.852	877.193	0.882
**XD03d**	0.994	1552	1559	4.694	26.115	0.638	0.946	11220	11259	7.891	942.507	0.883
**ZY01a**	0.993	1978	1991	5.231	65.419	0.703	0.943	11292	10875	7.934	982.318	0.878
**ZY01b**	0.993	1901	1862	5.223	55.701	0.672	0.938	10791	10559	7.909	966.184	0.890
**ZY01c**	0.989	1863	1830	5.125	52.972	0.675	0.933	10777	10558	7.850	791.139	0.885
**ZY01d**	0.992	2036	2062	5.213	65.432	0.669	0.922	10205	9961	7.872	925.069	0.885
**ZY02a**	0.991	1454	1471	4.871	47.375	0.668	0.943	10146	9789	7.774	839.631	0.869
**ZY02b**	0.993	1561	1602	4.931	49.940	0.670	0.921	8912	8713	7.727	874.126	0.884
**ZY02c**	0.995	1474	1495	4.887	48.821	0.673	0.939	10086	9859	7.792	883.392	0.864
**ZY02d**	0.994	1441	1467	4.850	45.769	0.677	0.934	9717	9516	7.746	879.508	0.872
**ZY03a**	0.991	1633	1655	5.007	39.569	0.670	0.928	9553	9346	7.760	885.740	0.923
**ZY03b**	0.993	1690	1714	5.078	51.565	0.685	0.949	10473	10311	7.822	885.740	0.866
**ZY03c**	0.992	1625	1606	5.004	49.778	0.685	0.935	9799	9600	7.743	729.395	0.868
**ZY03d**	0.991	1583	1584	4.915	37.600	0.687	0.935	10407	10193	7.792	846.740	0.867

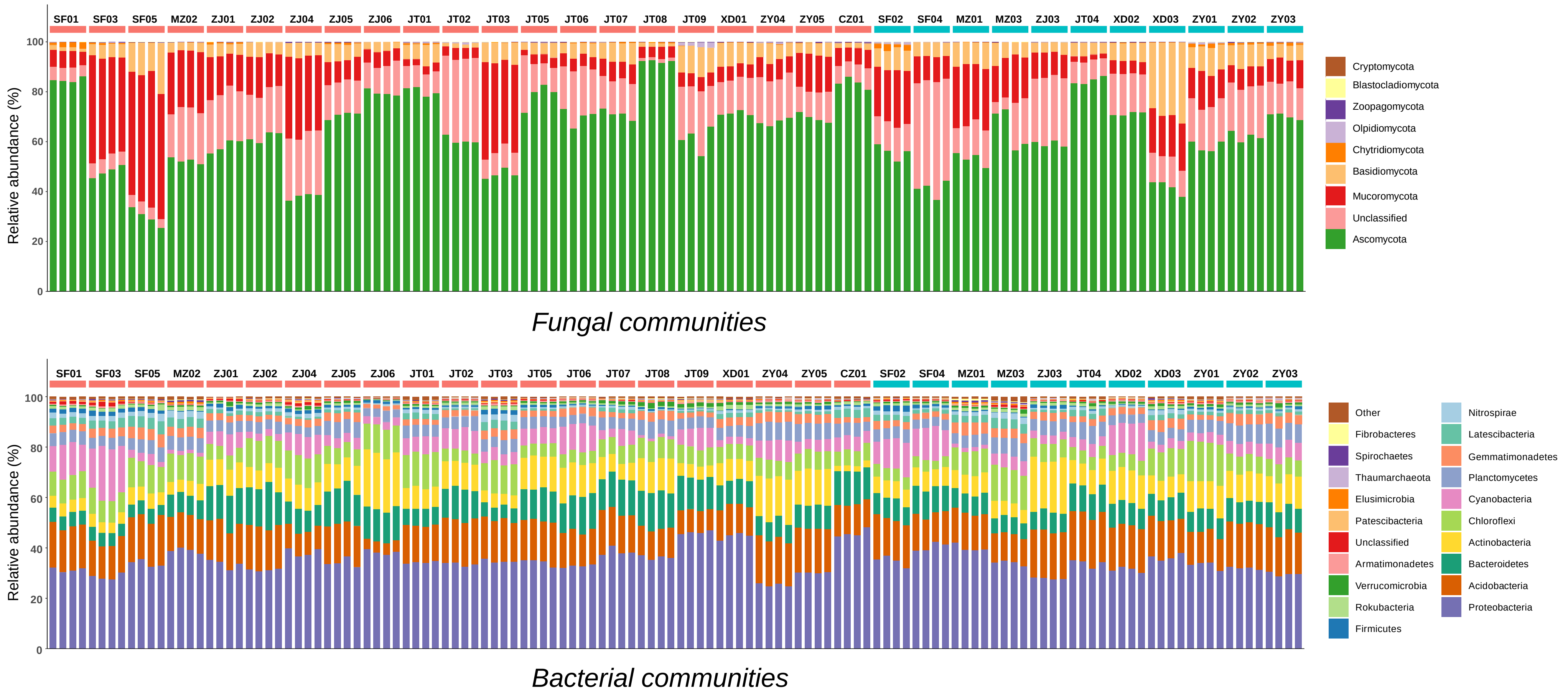

Fig. S1. Barcharts of community position at phylum level. Red tags below the site names mean non-fructification sites, while green means fructified sites.

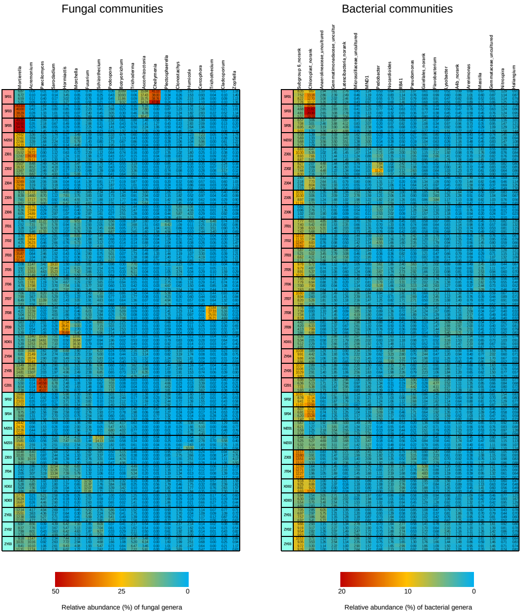

Fig. S2. Heatmaps of the composition of the fungal and bacterial communities at genus level, showing the 20 most prominent genera (top20).

7

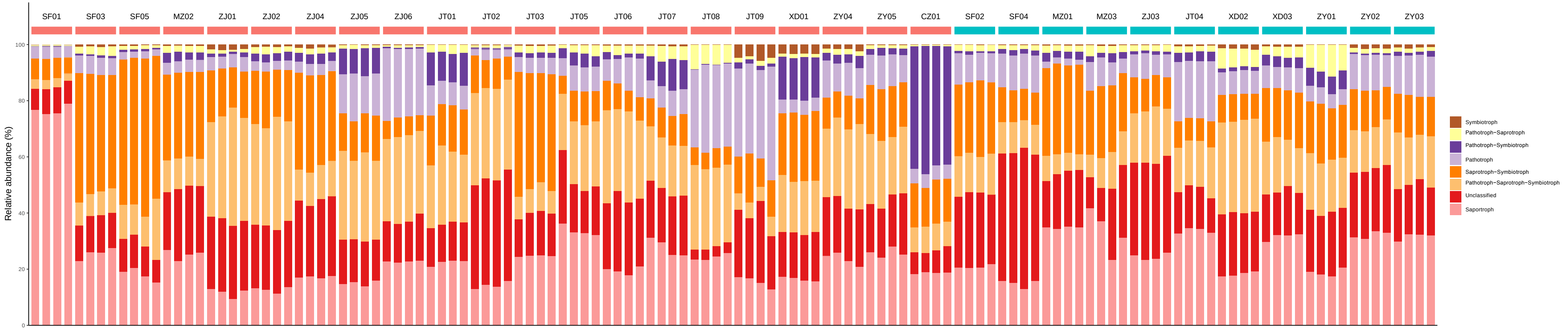

Fig. S3. Barchart of the composition of *in silico* predicted trophic modes in the fungal communities. Since the morel (*Morchella* spp.) as well as the *Mortierella* spp,, *Acremonium* spp., *Paecilomyces* spp., *Sarocladium* spp., and *Trichothecium* spp. predominant in the fungal communities of the non-fructification samples were classified into the confidence ranking of probable and possible, we used all the predictions ranked as highly probable, probable, and possible.

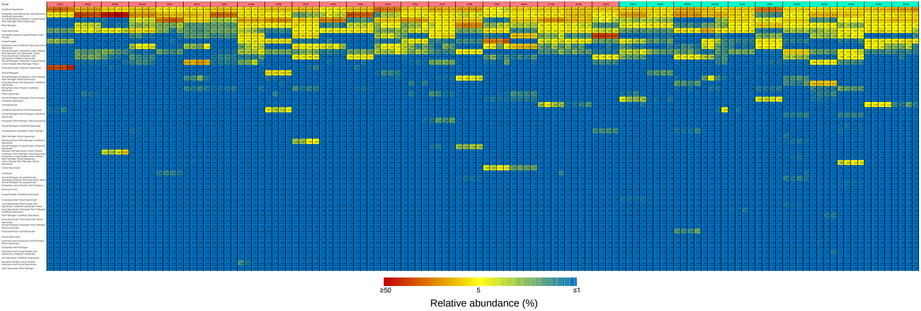

Fig. S4. Heatmap of the relative abundances of *in silico* predicted fungal guilds.

9

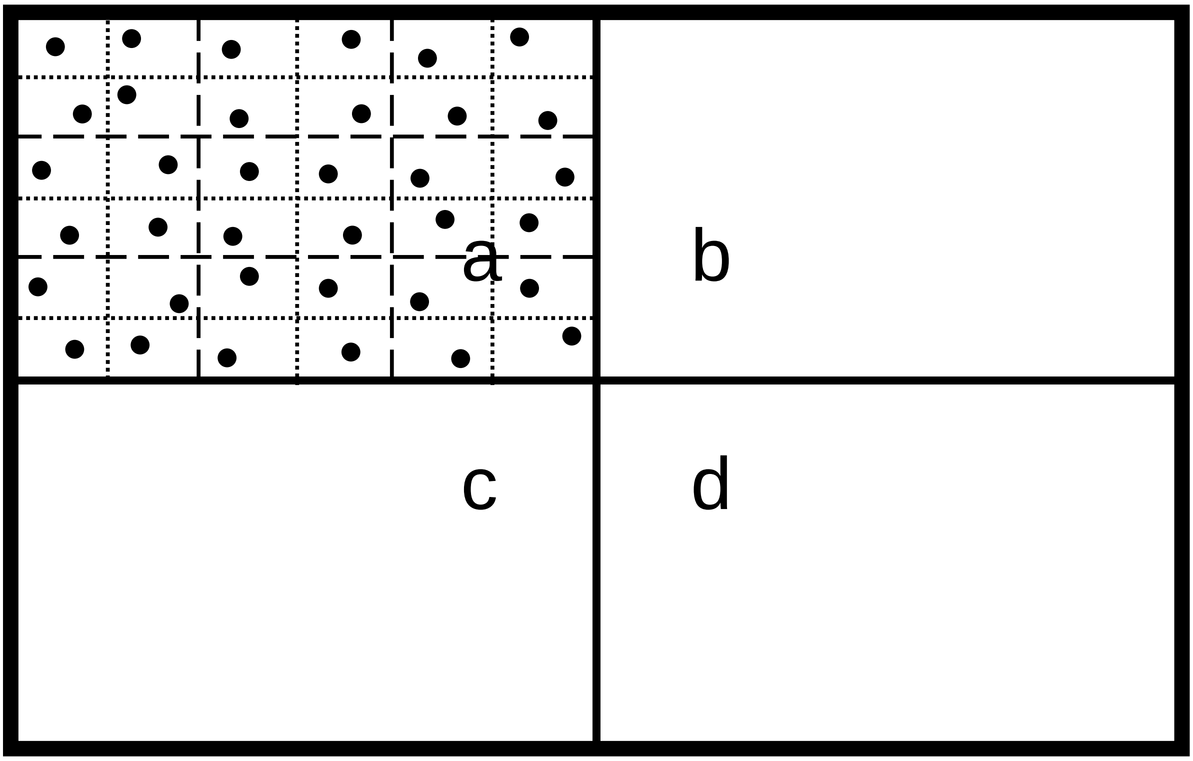

Fig. S5. Plot division and sampling strategy. Each investigated farm was divided into four plots: a, b, c, and d. In each plot, a "#" was drawn to divide the plot into nine equal aliquots. Each aliquot was further divided by a smaller cross into four equal grids. Eventually, each plot was divided into 36 small grids like a chessboard. A core of topsoil (in a cylinder shape, 5 cm diameter and 5 cm height) of mushroom-bed was taken from a random position in every small grid. Details in the "a" plot was shown, and the sampling strategy for each plot was identical.

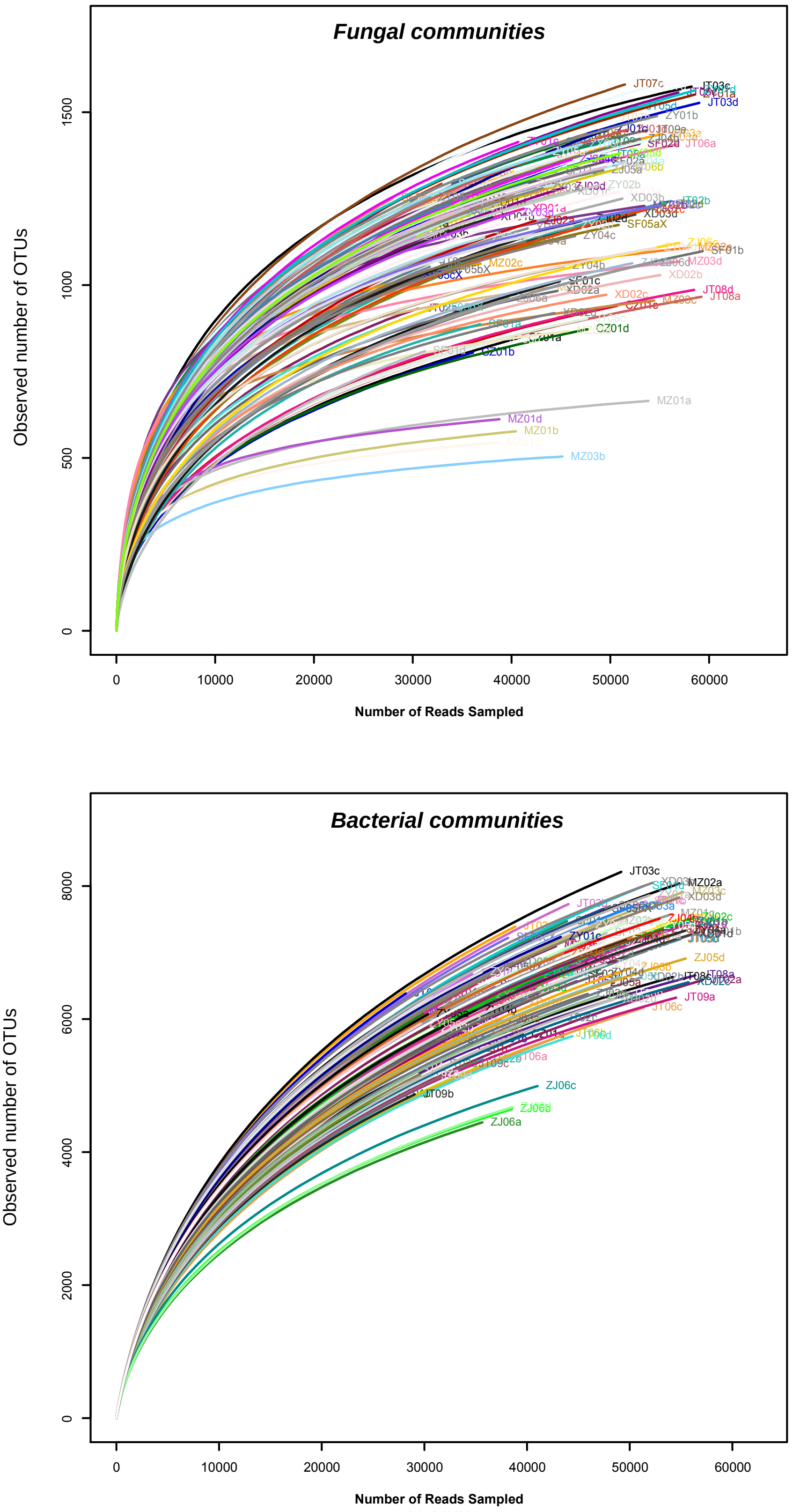

Fig. S6. Rarefaction curves of observed OTUs in the fungal and bacterial communities.
